# ‘Bud to fruit’—hormonal interactions governing early fruit development

**DOI:** 10.1093/jxb/eraf363

**Published:** 2025-08-14

**Authors:** Ranjit Baral, Andrii Vainer, Siegbert Melzer, Bettina Hause, Sayantan Panda

**Affiliations:** Department of Cell and Metabolic Biology, Leibniz Institute of Plant Biochemistry, Halle (Saale), Germany; Department of Plant and Environmental Sciences, Weizmann Institute of Science, Rehovot, Israel; Gilat Research Center, Agricultural Research Organization (ARO), Rural delivery Negev, Israel; Botanical Institute, Christian-Albrechts-University of Kiel, Kiel, Germany; Department of Cell and Metabolic Biology, Leibniz Institute of Plant Biochemistry, Halle (Saale), Germany; Department of Cell and Metabolic Biology, Leibniz Institute of Plant Biochemistry, Halle (Saale), Germany; University College Dublin, Ireland

**Keywords:** Abscisic acid, auxin, brassinosteroids, crosstalk, cytokinin, ethylene, fruit set, gibberellins, jasmonic acid, salicylic acid

## Abstract

Hormones are fundamental architects of plant reproduction, orchestrating the transition from pollination to fruit set. Recent advances have underscored the central roles of auxin and gibberellins in ovary growth, seed development, and parthenocarpy, while their intricate interplay with cytokinin, ethylene, abscisic acid, brassinosteroids, jasmonic acid, and salicylic acid fine-tunes early fruit development. A dynamic regulatory network involving transcription factors, miRNAs, and hormone-responsive genes modulates these processes, ensuring coordinated cellular events across diverse fruit types. Despite significant progress, the complexity of hormonal interactions and their species-specific nuances remain areas of active research. This review consolidates current insights into hormone-mediated fruit set, unraveling key molecular pathways and outstanding questions, with a focus on improving fruit production and crop resilience through targeted agricultural interventions.

## Teaser

This review highlights how plant hormones orchestrate early fruit development after pollination, revealing key molecular pathways that control fruit initiation and offering insights to improve crop productivity and stress resilience.

## Introduction

**Fig. 1.**
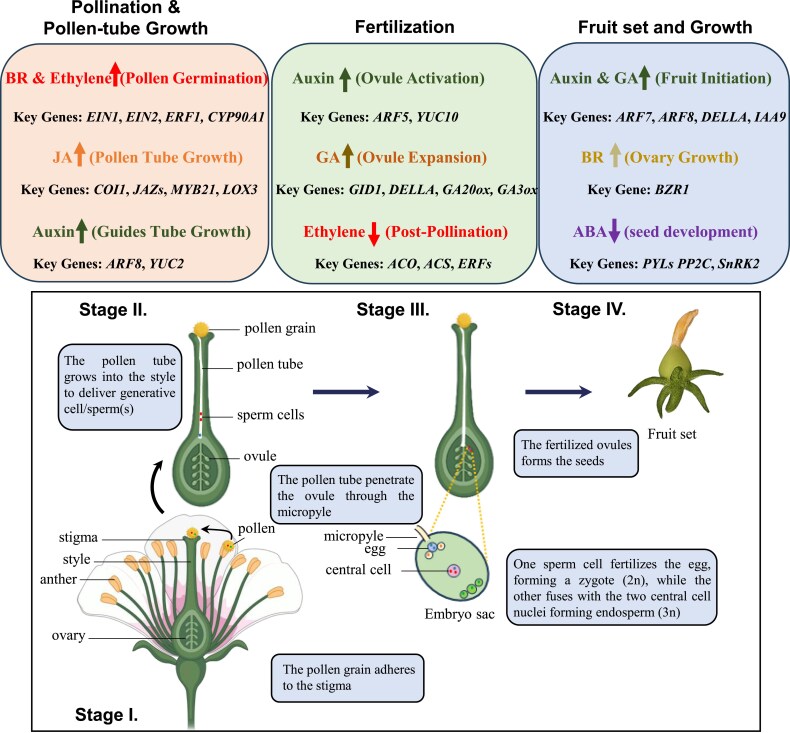
Schematic representation of key molecular players of specific hormones involved in progressive fruit development including pollen germination (stage I), pollen tube elongation (stage II), fertilization (stage III), and fruit set (stage IV). Ethylene signaling components, including ETHYLENE INSENSITIVE (EIN1 and EIN2) and the response factor ETHYLENE RESPONSE FACTOR (ERF1), are activated to facilitate pollination, followed by a sharp decline in ethylene levels post-pollination and fruit set, concomitant with the down-regulation of its biosynthetic enzymes 1-aminocyclopropane-1-carboxylic acid (ACC) synthase (ACS) and ACC oxidase (ACO). The brassinosteroid (BR) biosynthetic gene CYP90A1 enhances pollen germination and tube growth, while BR signaling via BRASSINAZOLE RESISTANT1 (BZR1) regulates ovary maturation. Jasmonic acid (JA) biosynthesis, mediated by LIPOXYGENASE (LOX3), and its signaling components, including the CORONATINE-INSENSITIVE 1–JASMONATE ZIM DOMAIN (COI1–JAZ) complex, MYC2, and MYB21, influence pollination, pollen tube growth, and seed development. Auxin positively regulates pollen tube growth, ovule activation, and fruit initiation through the activation of YUCCA (YUC) biosynthetic enzymes and AUXIN RESPONSE FACTORS (ARF7, ARF8), as well as the down-regulation of negative regulators such as INDOLE ACETIC ACID9 (IAA9). Gibberellin (GA) promotes ovule expansion and fruit initiation via biosynthetic enzymes (GA20ox and GA3ox) and signaling components (GID1 and DELLA proteins). Abscisic acid (ABA) regulates seed development and dormancy through its signaling module (PYR1–PP2C–SnRK2). PYL, PYRABACTIN RESISTANCE LIKE; PP2Cs, the group A type 2C protein phosphatases; SnRK2, SNF1-related protein kinases 2 (SnRK2s). Activation is indicated with a upward-pointing arrow (↑), while inhibition is indicated with a downward-pointing arrow (↓). Created in BioRender. Panda, S. (2025) https://BioRender.com/i58tlw6.

Development of floral organs (i.e. sepal, petal, stamen, and carpel) is to a large extent controlled by hormones including, auxin, gibberellins (GAs), cytokinin (CK), ethylene, abscisic acid (ABA), jasmonic acid (JA), salicylic acid (SA), and brassinosteroids (BRs) (Martínez *et al.*, 2004; Davis, 2009; Campos-Rivero *et al.*, 2017; Ito *et al.*, 2018; Schubert *et al.*, 2019; Wybouw and De Rybel, 2019; Li and He, 2020; Cucinotta *et al.*, 2021; Luo and Liu, 2022; Zhao *et al.*, 2022). Among these organs, the carpel, which matures into the fruit, serves as the primary site where hormonal interactions guide the transition from floral to fruit development. Notably, the early stages of fruit formation—regardless of whether the fruit is dry or fleshy—share common developmental cues. During successful reproductive development, the female reproductive organ of the flower—the gynoecium, and specifically its ovary—undergoes extensive growth and patterning to facilitate fruit initiation. The ovary produces ovules, which house the female gametophytes; within these, the egg cell (female gamete) is fertilized, initiating embryo development. After the ‘double fertilization’, the endosperm nucleus, originating from the fusion of a sperm cell and the two nuclei of the central cell, undergoes multiple mitotic divisions (Bleckmann *et al.*, 2014). This leads to commencement of growth in the ovary which progresses through rapid cell division, cell enlargement, and the generation of intracellular voids, ultimately resulting in fruit set (Gillaspy *et al.*, 1993). Apart from the ovary, in many cases, other tissues (e.g. the receptacle in apple, strawberry, and other *Rosaceae* members) grow and expand after fertilization, which lead to development of false fruits (Liu *et al.*, 2020; Yao *et al.*, 2021). Numerous hormones play prevalent roles in development of siliques in Arabidopsis, such as auxin, CK, GAs, and BRs, which all act on gene regulatory networks mediating gynoecium initiation and fruit development (Herrera-Ubaldo and de Folter, 2022). Similarly, in fleshy fruit development (e.g. in tomato), hormones play an important and unique role by regulating the transcription of various developmental pathways (Fenn and Giovannoni, 2021). For instance, auxin signaling, mediated by auxin response factors (ARFs) and Aux/IAA proteins, regulates early fruit development by activating genes involved in fertilization (e.g. *MADS-box* genes) and cell expansion (e.g. *EXPANSIN* genes) (Hu *et al.*, 2023), while GA signaling interacts with auxin pathways to promote fruit growth through DELLA protein degradation and modulation of GA-responsive genes (He and Yamamuro, 2022). Hormonal regulation of cell division and expansion is crucial in early fruit development (Fig. 1). A recent review by He and Yamamuro (2022) summarized the interaction between auxin and GA signaling pathways in horticultural plant models, including tomato and strawberry, to regulate the intricate balance between auxin and GA metabolism during early fruit development. The role of auxin in early fruit set was evaluated 30 years ago by Gillaspy *et al*. (1993) . Exogenous application of auxin and GAs enhances fruit set in the absence of fertilization, leading to parthenocarpy (fruit development without fertilization) (McAtee *et al.*, 2013; Kumar *et al.*, 2014). In the last decade, numerous pieces of evidence have been provided for auxin-mediated induction of other phytohormones, such as GAs, which coordinate fruit growth and development; however, their intricate crosstalk is not fully understood to date (He and Yamamuro, 2022). While auxin and GAs are known to be two major hormones for fruit growth (Ludwig-Müller, 2022), CK has been reported to be involved in the regulation of pericarp thickness and fruit size in fleshy fruits by promoting cell division during early stages of fruit development (Fenn and Giovannoni, 2021; Gan *et al.*, 2022). Genetic studies in tomato have shown that reducing endogenous CK levels through overexpression of a CK-inactivating enzyme from Arabidopsis (*At*CKX2) specifically in fruit tissues leads to a significant decrease in pericarp thickness and fruit size, primarily due to reduced cell number and lower expression of cell division-related genes (Gan *et al.*, 2022). Conversely, high concentrations of CKs during the period of active cell division have been detected in various fruits, including tomato and grape, supporting their positive role in early fruit growth and tissue expansion (Chacko *et al.*, 1976; Matsuo *et al.*, 2012; Böttcher *et al.*, 2015).

Transcriptomic studies have further elucidated the dynamic regulation of hormone-responsive gene networks that control cell division, expansion, and tissue differentiation during early fruit development (Pattison *et al.*, 2015; Zhang *et al.*, 2016; Shinozaki *et al.*, 2018; Ochoa-Alejo *et al.*, 2024). In tomato, RNA-seq analyses have highlighted key hormonal regulators—including ARFs, GA biosynthetic/signaling genes (*GA20ox*, *DELLA*), and the ABA biosynthesis gene *9-CIS-EPOXYCAROTENOID DIOXYGENASE* (*NCED1*)—as critical components governing ovary growth and the initiation of fruit set (Azzi *et al.*, 2015; Kai *et al.*, 2019). Beyond transcriptional regulation, post-transcriptional control by miRNAs)—such as *miR160*—has been shown to fine-tune hormone signaling pathways in species such as tomato and Arabidopsis by targeting ARFs and other transcription factors (TFs), thereby modulating the intensity and specificity of developmental responses (Damodharan *et al.*, 2016; Hao *et al.*, 2022). These multilayered regulatory mechanisms highlight the complexity of hormone-mediated fruit development and underscore the importance of integrative approaches to better understand their coordination and harness them for crop improvement and stress resilience.

Although extensive reviews have explored the hormonal regulation of floral transition and organogenesis through transcriptional, post-transcriptional, and epigenetic control mechanisms (Marsch-Martínez *et al.*, 2012; Yuan and Zhang, 2015; Campos-Rivero *et al.*, 2017; Conti, 2017; Izawa, 2021; Huang *et al.*, 2023), a comprehensive review on the hormonal control of post-fertilization events, early fruit set, and fruit development was published more than a decade ago (Obroucheva, 2014). Given the rapid advancements in our understanding of hormone-mediated fruit development, an updated survey of recent discoveries is essential. This review addresses this gap by providing an in-depth analysis of the latest insights into hormone-regulated fruit set. Unlike previous reviews that primarily focus on hormonal roles in fruit ripening (Ozga and Reinecke, 2003; Seymour *et al.*, 2013; Kumar *et al.*, 2014; Forlani *et al.*, 2019; Fenn and Giovannoni, 2021; Sharif *et al.*, 2022), this work explores the intricate hormonal crosstalk orchestrating fruit initiation. We highlight key molecular regulators, transcriptional networks, and hormone interactions governing pollination, fertilization, and fruit set, focusing on model species such as tomato (a fleshy fruit) and Arabidopsis (a dry fruit). By integrating recent advances, this review not only enhances our understanding of fruit set regulation but also lays the groundwork for future research and potential applications in crop improvement and sustainable fruit production.

## Auxin and gibberellic acid in pollination, fertilization, and fruit set

Fruit development is a highly coordinated process that begins with pollination, fertilization, and fruit set. Critical to this transition is the synchronized development of male and female reproductive organs—pollen grains and ovules—both of which rely heavily on the homeostasis and perception of the phytohormones auxin and GA. Microsporogenesis and pollen grain formation must align precisely with ovary and ovule development to ensure successful fertilization. The GAI–RGA–SCR (GRAS) TFs are a plant-specific TF family that derive the name from three members, GIBBERELLIN-ACID INSENSITIVE (GAI), REPRESSOR of GA1 (RGA), and SCARECROW (SCR), and play pivotal roles in modulating auxin and GA signaling (Khan *et al.*, 2022). These TFs regulate key developmental stages such as pollen sac development, pollen viability, and early fruit set. In tomato, for instance, the GRAS24 TF is known to be a negative regulator of early fruit set that is inhibited prior to pollen maturity (Huang *et al.*, 2017). This is facilitated by *miR171*, which post-transcriptionally regulates *GRAS24* to maintain GA and auxin homeostasis (Huang *et al.*, 2017). Besides *miR171*, numerous other miRNA regulatory modules were found to interplay with hormone networks to secure plant reproduction (Correa *et al.*, 2018). Recently, the *miR166*–*Sl*HB15A module that post-transcriptionally controls ovule development and fruit set in the absence of fertilization has been identified in tomato (Clepet *et al.*, 2021). In this pathway, *miR166* targets and down-regulates the expression of the class III HD-Zip TF *Sl*HB15A, which is specifically expressed in the ovule integument. Mechanistically, *Sl*HB15A acts as a bifunctional regulator: it represses auxin signaling by binding to the promoters of auxin-related genes (*TOMATO FLOOZY 2/3*, *PIN4,* and *ARF7*) and activates ethylene signaling by binding to the promoters of ethylene-related genes [*EIL2*, *ACC oxidase 4* (*ACO4*), and *ERF* genes] in ovules (Clepet *et al.*, 2021). By repressing *Sl*HB15A, *miR166* modulates both auxin and ethylene signaling pathways within ovule tissues, thereby promoting proper ovule development and enabling parthenocarpic fruit set, particularly under environmental stress conditions such as high temperature (Clepet *et al.*, 2021).

**Fig. 2.**
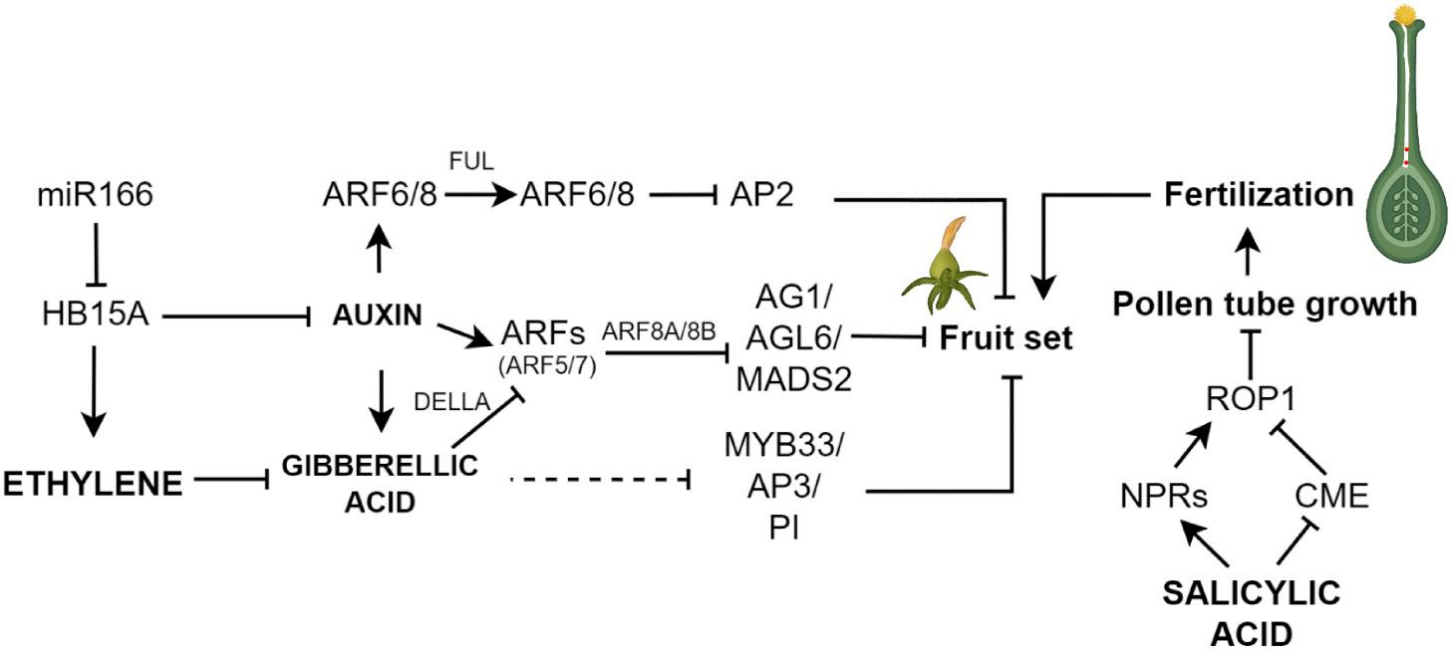
Schematic representation of the interaction between diverse hormone pathways and their roles in pollen tube growth, fertilization, and fruit set, with special emphasis on a fleshy fruit model such as tomato. Broken lines indicate indirect manipulation of a process or unknown mechanism. In tomato, miR156 mediates regulation of the HOMEOBOX 15A (*Sl*HB15A) TF which modulates auxin and ethylene signaling to initiate fruit set. Auxin Response Factors (ARFs) acts as central TFs for interlinking auxin and GA to influence MADS-box genes such as *AGAMOUS* (*AG*) to determine fruit set. GA regulates fruit set via a DELLA-dependent pathway. SA influences pollen tube growth and subsequent fertilization through its master regulator *NPR*, which activates pollen-specific ROP1 (Rho-related in Plant), a unique subfamily of Rho-GTPases in plants. Furthermore, SA antagonistically regulates pollen tube growth through CLATHERIN-MEDIATED ENDOCYTOSIS (CME). AP2/3, APETALA 2/3; PI, PISTILLATA; FUL, FRUITFUL. Activation is indicated with an arrow (→), while inhibition indicated with a blunted arrow (┴). Created in BioRender. Panda, S. (2025) https://BioRender.com/jx1i1tu.

Alongside hormonal regulation, fruit set also requires profound metabolic reprogramming. In tomato, Shinozaki *et al.* (2020) revealed that GA signaling—through its repressor DELLA/PROCERA—drives a major transition in central metabolism during fruit set. Their multi-omics study showed enhanced sugar mobilization, increased fructokinase activity, and activation of tonoplastic sugar transporters to meet the biosynthetic and energetic demands of ovary growth. This comprehensive analysis also uncovered elevated levels of hexoses, sugar phosphates, and key intermediates of glycolysis and the tricarboxylic acid (TCA) cycle in fruit-setting ovaries. Within this regulatory framework, *Sl*HB15A was identified as a key transcriptional integrator linking auxin and ethylene signaling (Fig. 2) with metabolic activation and cell wall biosynthesis (Shinozaki *et al.*, 2020), reinforcing its centrality in early fruit development.

Moreover, the *KNOTTED-like homeobox gene 4* (*TKN4*) has been identified as a key regulator that enhances the sensitivity of the plant to both auxin and GA, thereby facilitating pollen germination, fruit set, and seed development in tomato (Yan *et al.*, 2019). In *TKN4*-overexpressing tomato lines, several GA-related genes (*SCL3*, *SCL32*, *GRAS6*, *GA20ox2*, and *GA2ox5*) and auxin-related genes (*PIN3*, *PIN9*, *GH3.8*, and *ARF9*) were significantly up-regulated. These findings align with the well-established role of GAs in mediating the developmental switch from flowering to fruit initiation, as GA levels naturally rise in the ovary upon pollination (Serrani *et al.*, 2007; Dorcey *et al.*, 2009). Moreover, exogenous application of GA to flowers can induce parthenocarpic fruit formation even in the absence of fertilization (Gorguet *et al.*, 2005). In tomato, GA treatment-induced parthenocarpy is mediated by suppression of the ARF auxin signaling components, such as *Sl*ARF5 and *Sl*ARF7, and the MADS-box genes *APETELLA3* (*AP3*), *PISTILLATA*, and *AGAMOUS-LIKE6* (*AGL6*) (Niu *et al.*, 2023). Characterization of the GA-insensitive *gib1* mutant (mutant for one of the GA receptors), revealed that auxin-induced tomato fruit initiation and parthenocarpy are GA dependent (Hu *et al.*, 2018). In this work direct protein–protein interactions have been identified between *Sl*DELLA proteins and *Sl*ARF7, which serve as mechanistic crosstalk between auxin and GA signaling during tomato fruit development (Hu *et al.*, 2018). Similarly, applying exogenous GA or mutating the GA signaling repressor RGA1, a DELLA protein, was found to induce parthenocarpy in woodland strawberry (*Fragaria vesca*) (Kang *et al.*, 2013; Zhou *et al.*, 2021). RGA1 directly interacts with ARF8 in woodland strawberry, and *Fvarf8* mutant fruits displayed increased sensitivity to both auxin and GA application, accompanied by elevated expression of *FvGID1c* (*GA-INSENSITIVE DWARF1*), a GA receptor. This underscores *Fv*ARF8-mediated crosstalk between auxin and GA signaling pathways (Zhou *et al.*, 2021).

GA-induced parthenocarpy extends across species. In pear (*Pyrus bretshneideri*), exogenous GA application was found to trigger parthenocarpy through induction of *PbCYP78A6*, an Arabidopsis *EOD3/AtCYP78A6* ortholog, known to control reproductive organ development though an as yet unknown catalytic function (Anastasiou *et al.*, 2007; Fang *et al.*, 2012; Qi *et al.*, 2019). Likewise in grapes (*Vitis vinifera*), exogenous GA application is known to induce parthenocarpy, through *miR159c* mediated post-transcriptional regulation of *Vv*GAMYB (Wang *et al.*, 2018), a TF that might interact with *AINTEGUMENTA (ANT)-like* genes for successful ovule development (da Silva *et al.*, 2017).

In addition to GA, auxin plays prevalent roles in initiating fruit set across various plants (de Jong *et al.*, 2009; Liu *et al.*, 2018; Zhang *et al.*, 2021). During early fruit set of watermelon, in both regularly pollinated and parthenocarpic fruits, the level of auxin and GA increased noticeably, followed by a sharp increase in expression of auxin-responsive genes (Hu *et al.*, 2019). Similarly in grapes, the role of auxin in the molecular control of early fruit development has been noticed (Godoy *et al.*, 2021). Temporal transcriptomic and hormone profiling studies have shown that auxin levels and related gene expression dynamically change during the early stages of fruit set. Matsuo *et al.* (2018) demonstrated that indole-3-acetic acid (IAA) and its precursor indole-3-pyruvic acid (IPyA) increase in tomato ovaries shortly after pollination, along with up-regulation of key biosynthetic genes (*TRYPTOPHAN AMINOTRANSFERASE RELATED 2* and *FLAVIN MONOOXYGENASE-LIKE YUCCA 1/2/5*), indicating that auxin biosynthesis via the IPyA pathway drives fruit initiation. In contrast, auxin-inactivating genes (*SlGH3-9* and *SlGH3-15*) are elevated in unpollinated ovaries but decline after pollination. Consistent with this pattern, exogenous application of synthetic auxin (2,4-D) mimics pollination effects, reinforcing the role of auxin in sustaining its own accumulation. This finding is further supported by Godoy *et al.* (2021), who showed that treatment with an exogenous auxin inhibitor (i.e. IAA-Trp) reduced cell number and mesocarp diameter in grape berries, closely resembling the developmental traits of unpollinated fruit.

A central node in auxin-mediated suppression of fruit initiation is mediated by MADS-box TFs, where they act to inhibit parthenocarpy by repressing developmental pathways that trigger ovary growth without fertilization. Earlier studies demonstrated that applying auxin or GA to tomato flowers leads to the post-fertilization transcriptional suppression of *SlAGL6* (*AGAMOUS-LIKE6*; encoding a MADS-box TF) (Wang *et al.*, 2009; Pattison *et al.*, 2015; Tang *et al.*, 2015). Furthermore, *Sl*AGL6 was identified as a key regulator of fruit set, and silencing of *SlAGL6* leads to the release of the ‘ovary arrest’ state, resulting in the formation of parthenocarpic fruit in tomato (Klap *et al.*, 2017). In tomato, *SlAGL6* is predominantly expressed in the integument of the immature ovule and, upon ovule maturation, its expression shifts to the endothelium (Gupta *et al.*, 2021). *Sl*AGL6 inhibits parthenocarpic fruit set through reprogramming of ovule integument, facilitated by the growth regulator *Sl*KLUH, a fertilization-induced cytochrome P450 that regulates cell proliferation (Gupta *et al.*, 2021). Furthermore, recent studies have uncovered the transcriptional regulation of *SlAGL6* by the ARF TFs *Sl*ARF8A and *Sl*ARF8B (Hu *et al.*, 2023; Israeli *et al.*, 2023). In tomato, individual mutants (*Slarf8a* and *Slarf8b*) display partial parthenocarpy, whereas the double mutant (*Slarf8a×Slarf8b*) shows complete development of parthenocarpic fruit. In depth molecular characterization revealed that ARF8A and ARF8B physically interact with IAA9 and regulate MADS-BOX TFs [AGAMOUS1 (AG1), MADS2, and AGL6] to inhibit fruit set (Hu *et al.*, 2023; Israeli *et al.*, 2023) (Fig. 2). Transgenic tomato plants overexpressing truncated *Sl*ARF8A/8B (lacking the IAA9-interacting PB1 domain) demonstrated robust parthenocarpy, providing additional evidence for *Sl*ARF8A/8B-mediated fruit growth. Moreover, RNA-seq coupled with ChIP and quantitative PCR analyses identified the target genes of *Sl*ARF8A/8B, including those encoding the MADS-BOX TFs, which act as crucial suppressors of fruit set. These findings suggest that in the absence of the auxin-dependent ARF8A*/*ARF8B–IAA9 interaction, parthenocarpic fruit set is promoted (Hu *et al.*, 2023; Israeli *et al.*, 2023). Like tomato *AGL6*, both in diploid strawberry and in Arabidopsis, a type I MADS box gene *AGL62* was found to activate auxin synthesis in the endosperm (Guo *et al.*, 2022). Additionally, *SEPALLATA* (*SEP*), class E homeotic genes in flower development, also take part in controlling early stages of fruit development in Arabidopsis through transcriptional targeting of several components involved in GA and auxin metabolism/signaling, including ARF TFs (ARF3, ARF6, and ARF8), GRETCHEN HAGEN3 (GH3.3), PIN4, and PINOID (PID) (Kaufmann *et al.*, 2009). In tomato, reduced expression of *TEMPRANILLO* (*TM29*/*SEP1/2*-like) and *TM5* (*SEP3*-like) leads to parthenocarpy (Pnueli *et al.*, 1994; Ampomah-Dwamena *et al.*, 2002). A similar observation was made in apple, where a transposon insertion in the introns of a *SEP* gene induced parthenocarpy (Yao *et al.*, 2001). Recently, a class E gene homolog *eSEP3* was characterized in strawberry which is induced only in pollinated flowers 6–7 d after anthesis and is co-expressed with auxin pathway genes, suggesting a possible involvement in auxin-mediated fruit set (Pi *et al.*, 2021) as well as the involvement of class E genes as repressors of fruit development in the absence of fertilization.

Taken together, these findings present a nuanced and integrated picture of how auxin and GA signaling converge at multiple regulatory nodes—TFs, miRs, and hormone receptors—to tightly coordinate pollen maturation, ovule development, fertilization, and fruit initiation. This coordination ensures a timely developmental switch from flowering to fruit set, and its modulation underlies species-specific differences in reproductive strategies. The conservation of regulatory modules such as DELLA–ARF, *miR171*–GRAS24, and *miR166*–*Sl*HB15A across species underscores the evolutionary robustness of these networks. Importantly, the discovery of hormone-responsive transcriptional reprogramming and crosstalk with metabolic activation during early fruit development reveals potential targets for improving fruit set under stress conditions.

## Abscisic acid and ethylene regulate pollination and fruit set

ABA and ethylene are central regulators of reproductive development, playing critical roles from pollination to fruit set. ABA acts primarily as a growth inhibitor, while ethylene mediates tissue differentiation and senescence. Both hormones engage in dynamic crosstalk with other hormonal signals such as auxin and GA, and this hormonal network is tightly integrated with developmental and environmental cues to coordinate reproductive success.

The ABA pathway is defined by three core components: the ABA receptor proteins PYRABACTIN RESISTANCE (PYR)/PYR-LIKE (PYL)/REGULATORY COMPONENT OF ABA RECEPTOR (RCAR), which perceive ABA and inhibit group A type 2C protein phosphatases (PP2Cs), thereby releasing subclass III SNF1-related protein kinases 2 (SnRK2s) to activate downstream ABA responses (Ma *et al.*, 2009; Melcher *et al.*, 2009; Park *et al.*, 2009). This canonical module is highly conserved and underpins the regulatory roles of ABA in fruit development and maturation across multiple species (Leng *et al.*, 2018; Gupta *et al.*, 2022).

During pollination and early fruit set, ABA accumulates in the ovary and developing fruit, where it modulates gene expression to ensure proper reproductive progression. In sweet cherry (*Prunus avium*), high transcript levels of ABA-responsive genes such as *SNF1-RELATED PROTEIN KINASE 2* and *PP2C*, and *PYRABACTIN RESISTANCE 1-LIKE PROTEINS* have been detected in young fruit and ovaries, highlighting the positive influence of ABA on ovary maturation and fruit initiation (Wang *et al.*, 2015; Shen *et al.*, 2017; Leng *et al.*, 2018) (Fig. 1). In tomato, ABA biosynthesis and signaling are tightly linked to the regulation of fruit set, with *SlNCED1* acting as a key biosynthetic gene whose up-regulation is associated with successful fruit development (Sun *et al.*, 2012; Ji *et al.*, 2014). Beyond fruit development, *Sl*NCED1 also ensures reproductive success by regulating genes involved in pollen hydration, germination, and tube elongation—processes essential for pollen viability and fertilization (Dai *et al.*, 2018; Wang *et al.*, 2021). In Arabidopsis, the *ENHANCED RESPONSE TO ABA 1* (*ERA1*) gene, encoding the β-subunit of farnesyltransferase, is required for proper pollen germination and seed formation, as protein farnesylation modulates ABA sensitivity in reproductive tissues (Vergès *et al.*, 2021). Moreover in Arabidopsis, mutants deficient in ABA biosynthesis (*aba1*, *aba2*) or insensitive to ABA signaling (*abi3*, *abi4*, *abi5*, and the *snrk2.2 snrk2.3 snrk2.6* triple mutant) frequently exhibit defects in pollen function, reduced fertilization efficiency, and abnormal seed development (Söderman *et al.*, 2000; Fujii and Zhu, 2009; Cheng *et al.*, 2014; Skubacz *et al.*, 2016). At the molecular level, ABA signaling during pollination and fruit set is mediated by the activation of SnRK2s, which phosphorylate ABA-responsive TFs such as ABFs/AREBs, triggering the expression of downstream genes involved in cell expansion (e.g. *EXPANSIN* genes) and sugar transport/metabolism (e.g. *SWEET* genes and *AMYLASE*) (Wu *et al.*, 2023). In addition, ABA modulates seed development by negatively regulating the expression of *SHORT HYPOCOTYL UNDER BLUE1* (*SHB1*) via ABI5. ABI5 binds to the *SHB1* promoter, repressing its activity during early seed development (Cheng *et al.*, 2014). As a key positive regulator of seed growth, *SHB1* promotes endosperm proliferation and seed cavity expansion by directly activating *MINISEED3* (*MINI3*) and *HAIKU2* (*IKU2*), two key genes essential for proper endosperm formation and seed size determination (Luo *et al*., 2005; Zhou *et al.*, 2009).

The role of ABA in reproductive success is reinforced through interactions with ethylene, to fine-tune the timing of fruit set and development. For example, ABA can modulate ethylene biosynthesis and signaling, thereby influencing ovule longevity and the onset of fruit growth (Chai *et al.*, 2017; Fenn and Giovannoni, 2021). In tomato, both ABA and ethylene play pivotal, often antagonistic, roles in regulating the transition from ovary to fruit. Prior to anthesis in tomato, the ethylene biosynthesis enzyme 1-AMINOCYCLOPROPANE-1-CARBOXYLIC ACID SYNTHASE (ACC synthase) and the ETRs (ethylene receptors) are induced in pollen grains to maintain pollen germination and pollen tube growth (Kovaleva *et al.*, 2017; An *et al.*, 2020). A similar mechanism is observed in tobacco (*Nicotiana tabacum*), where ethylene emission from the stigma accelerates the thinning of transmission tissues, facilitating pollen tube growth and successful fertilization (De Martinis *et al.*, 2002). In parallel, ABA accumulates in the ovary before pollination, where it acts as a growth inhibitor to maintain ovary dormancy. Following pollination, in tomato, a decline in ABA biosynthesis—particularly through down-regulation of NCED1—and an increase in ABA catabolism led to a sharp reduction in ABA content, thereby lifting growth inhibition and allowing fruit set to proceed (Nitsch *et al.*, 2009). Simultaneously, ethylene biosynthetic genes (e.g. *ACO2* and *ACO4*) also show decreased expression following pollination (Llop-Tous *et al.*, 2000). However, a transient burst of ethylene occurs immediately after fertilization, driven by up-regulation of *ACC synthase* (*ACS*) and *ACO* genes in the ovary (Llop-Tous *et al.*, 2000; Pattison *et al.*, 2015) (Fig. 1). At the molecular level, ABA and ethylene crosstalk is mediated by TFs and signaling components. In tomato, the ZINC FINGER PROTEIN 2 modulates the expression of both ABA and ethylene biosynthetic genes, integrating signals from both pathways to coordinate fruit development (Weng *et al.*, 2015a). Moreover, ABA suppresses ethylene biosynthesis by down-regulating *ACS* and *ACO* genes, while ethylene, in turn, modulates ABA sensitivity and signaling via its receptors and downstream components (Nitsch *et al.*, 2009; Weng *et al.*, 2015a). This reciprocal regulation is exemplified in ABA-deficient tomato mutants such as *not/flc*, which display reduced ABA levels, smaller fruit cells, and heightened ethylene emission—highlighting the antagonistic interaction that balances ABA and ethylene signaling during early fruit development (Nitsch *et al.*, 2012).

Beyond coordinating fruit development, the transient ethylene burst in pollinated ovaries triggers the senescence and abscission of floral organs, facilitating resource reallocation to the developing fruit. In tobacco, this ethylene burst activates the key signaling component EIN2, which subsequently induces the expression of senescence-associated genes (SAGs) (Chakrabarti and Bharti, 2023). Ethylene-sensitive flower senescence has been documented across numerous plant species, with many SAGs identified as central players in this process (Dar *et al.*, 2021). In carnation (*Dianthus caryophyllus*), ethylene perception involves receptors such as *DcERS1*, *DcERS2*, and *DcETR1*, each showing specific expression patterns across developmental stages and floral organs. Notably, while *DcERS2* and *DcETR1* transcripts accumulate in petals at full bloom, *DcERS2* expression diminishes during senescence, and *DcERS1* mRNA is absent at all petal stages—indicating functional divergence among these isoforms (In *et al.*, 2013). This ethylene-driven regulatory cascade culminates in floral organ degradation, mediated by EIN2-induced expression of proteases and cell wall-modifying enzymes (Leslie *et al.*, 2007). Consistent with this, overexpression of *EIN2* in petunia accelerates petal senescence through elevated ethylene production, while pollination also induces expression of ethylene biosynthetic genes such as *ACO1* and various cysteine proteases that expedite tissue breakdown (Shibuya *et al.*, 2004). Similar mechanisms operate in Arabidopsis, where ethylene promotes senescence and abscission of floral organs. Loss-of-function mutations in key ethylene signaling genes (*ETR1* and *EIN2*) delay floral senescence and abscission, highlighting the essential role of ethylene (Chen *et al.*, 2015, 2022; Iqbal *et al.*, 2017). In contrast, mutations in CTR1 (CONSTITUTIVE TRIPLE RESPONSE1), a negative regulator of ethylene signaling, cause constitutive ethylene responses and result in premature floral organ senescence and abscission (Chen *et al.*, 2015; Iqbal *et al.*, 2017). Additionally, in Arabidopsis, the *FOREVER YOUNG FLOWER* gene (*FYF*; encoding a MADS-box TF) functions as a negative regulator of floral aging by suppressing ethylene response DNA-binding factors (EDFs), key downstream targets in the ethylene signaling pathway. Loss of FYF function leads to up-regulation of EDFs and accelerates floral senescence and abscission (Chen *et al.*, 2015, 2022). Together, these studies confirm the conserved and central role of ethylene as a master regulator of floral senescence, ensuring the timely dismantling of non-essential floral structures and efficient nutrient remobilization toward reproductive success.

Ethylene also interacts closely with ABA in regulating floral senescence, often in a synergistic manner. Both hormones independently promote the aging of petals and other floral organs, but their combined action can further amplify the intensity and pace of senescence. In rose, exogenous application of either ABA or ethylene accelerates petal senescence, with ABA enhancing ethylene sensitivity by up-regulating the expression of ethylene receptors, a process mediated by the *Rh*HB1 TF (a member of the homeodomain-leucine zipper I family TFs) (Müller *et al.*, 2000; Lü *et al.*, 2014). Likewise, in petunia, ABA or ethylene treatment alone advances flower wilting, while their combined application results in even faster senescence compared with controls, indicating additive or synergistic effects (Chang *et al.*, 2014). At the molecular level, this hormonal interplay is mediated *Ph*HD-Zip TF, which is activated by both ABA and ethylene to regulate downstream genes involved in ABA biosynthesis and ethylene signaling, thereby promoting floral senescence (Chang *et al.*, 2014).

Overall, the intricate interplay between ethylene and ABA orchestrates the precise timing of floral organ senescence and fruit development. This dynamic hormonal crosstalk ensures that reproductive processes are tightly regulated, allowing plants to optimize resource allocation, maximize reproductive efficiency, and adaptively respond to changing environmental conditions for successful seed and fruit set.

## Cytokinin and brassinosteroids in fertilization and fruit initiation

CKs are pivotal regulators of reproductive development, influencing fertilization, fruit set, and early fruit growth across diverse plant species. After fertilization, CK levels typically rise in developing ovaries, indicating their involvement in activating fruit initiation programs (Terceros *et al.*, 2020; Hussain *et al.*, 2025). Exogenous CK application has been shown to stimulate fruit set in several crops, such as cucumber (*Cucumis sativus*), where CK treatment significantly enhances fruit initiation and early growth (Su *et al.*, 2021). In strawberry (*F. vesca*), increased CK (*trans*-zeatin) content in fertilized achenes (the actual fruit) and up-regulation of CK-responsive genes after pollination further support the positive role of CK in fruit set (Tian *et al.*, 2022).

In Arabidopsis, CK signaling is essential for the growth of the medial domain of the gynoecium and the formation of valve margins during early fruit development (Marsch-Martínez *et al.*, 2012). CK-responsive reporter lines, such as *TCS:GFP* (two-component output sensor; contains six direct repeats of the cytokinin-induced B-type Arabidopsis response regulator-binding motif), have revealed strong CK activity in these tissues. Both exogenous CK application and genetic manipulation of CK pathways lead to pronounced alterations in fruit patterning and morphogenesis, highlighting its developmental significance (Marsch-Martínez *et al.*, 2012). Importantly, CK does not act alone; substantial evidence indicates that CK and auxin signaling are tightly coordinated during early fruit development. CK signaling is particularly active in proliferating cells of CK-treated gynoecia, often overlapping with auxin-responsive domains, as demonstrated by co-localization of CK and auxin reporter activity (Marsch-Martínez *et al.*, 2012; Tian *et al.*, 2022; Hussain *et al.*, 2025). CK treatment can also up-regulate auxin biosynthesis and signaling genes, and the full promotive effect of CK on cell proliferation is diminished in auxin signaling mutants, demonstrating that auxin is required for optimal CK-mediated growth (Müller *et al.*, 2017). Genetic studies in Arabidopsis reinforce this synergy, as mutations in either CK (*cre1-12*, *ahk2-2*, *ahk3-3*; *arabidopsis histidine kinase4/cytokinin response*) or auxin (*pin1-5*, *bel1-1*, and *spl-1*) pathways result in similar defects in ovule patterning, with double/triple mutants often displaying enhanced or additive phenotypes (Bencivenga *et al.*, 2012). A similar observation was made in tomato, where spatiotemporal transcriptomic analyses revealed that CK and auxin coordinately regulate the expression of genes involved in cell cycle progression and tissue differentiation during the early stages of fruit development (Pattison *et al.*, 2015). Additionally, CKs play a vital role in ovule, pollen, and seed development by modulating molecular pathways essential for reproductive success (Terceros *et al.*, 2020). At the molecular level, CKs have been shown to up-regulate genes such as *CYCLIN D3* and *SPATULA* [encoding a basic helix–loop–helix (bHLH) TF], both of which are involved in cell cycle regulation (Cerbantez-Bueno *et al.*, 2024), as well as type-A *ARR* genes, which function as feedback regulators of cytokinin signaling (To *et al.*, 2004). Collectively, these findings highlight that CK acts in close coordination with auxin to orchestrate the complex processes of cell division and tissue specification necessary for successful fruit initiation.

Besides CK, BRs have also been identified as key regulators of reproductive development, including fertilization, fruit set, and early fruit growth across various species. For example, exogenous BR application in walnut (*Juglans regia*) markedly promotes pollen tube elongation, facilitating sperm delivery to the ovule (Sotomayor *et al.*, 2022). In Arabidopsis, overexpression of the BR biosynthetic gene *CYP90A1* enhances pollen tube formation and increases fertilization efficiency (Vogler *et al.*, 2014) (Fig. 1). These findings underscore the importance of BRs in optimizing male gametophyte performance and fertilization success. In Arabidopsis, BRs regulate the expression of key genes involved in ovule development, such as *HUELLENLOS*, *AINTEGUMENTA* (*ANT*), and *APETALA2* (*AP2*), either directly by binding to their promoter regions or indirectly through the BR-responsive TF BZR1 (BRASSINAZOLE RESSISTANT1, a signaling component of BR) (Huang *et al.*, 2013). In addition to regulating ovule development, the BZR1-like TF (BZR1.7) promotes tomato fruit elongation by binding to the conserved E-box in the *SUN* gene promoter and enhancing its expression, as SUN plays an essential role in determining fruit shape (Yu *et al.*, 2022). Furthermore, BR-deficient (*det2*) or BR-insensitive (*bri1-5*) mutants exhibit reduced ovule production and lower seed set, while exogenous application of BRs restores normal reproductive development (Jiang and Lin, 2013; Baghel *et al.*, 2019). Similar regulatory roles have been observed in fruit crops such as tomato, cucumber, and persimmon (*Diospyros kaki*), where BRs positively influence both ovule number and seed yield (Fu *et al.*, 2008; Barro-Trastoy *et al.*, 2020; Li *et al.*, 2024). In cucumber, transcriptomic and physiological studies have demonstrated that BRs are essential for the initiation of fruit development by promoting active cell division and up-regulating cell cycle-related genes, particularly *CycD3;1* and *CycD3;2* (*cyclin D3* genes) (Fu *et al.*, 2008). Application of exogenous BRs (such as 24-epibrassinolide) induces parthenocarpic fruit growth and increases the expression of these cyclins, while inhibition of BR biosynthesis represses their expression and prevents fruit set (Fu *et al.*, 2008). Mechanistically, BR signaling in cucumber probably involves homologs of the BZR1 TF, as seen in other species: BZR1 and BES1 are key downstream effectors of BR signaling in Arabidopsis (Kono and Yin, 2020). Recent studies further demonstrate that BZR1/BES1 play critical roles in reproductive development by directly regulating genes associated with ovule development and early differentiation of fruit tissues in different species. In Arabidopsis, the gain-of-function *bzr1-1D* mutant produces significantly more ovules and seeds, while BR-deficient (*det2-1*) mutants show reduced reproductive output (Fujioka *et al*., 1997; Huang *et al*., 2013). A similar correlation between BR levels and ovule number has been reported in tomato, where BR-enriched genotypes (e.g. MT-*D*) produce more ovules than the wild type [Micro-Tom (MT)], and exogenous BR treatment increases ovule number in MT, effectively complementing its lower endogenous BR content (Barro-Trastoy *et al*., 2020). These findings support a conserved role for BRs in promoting ovule primordia formation across species. However, unlike in Arabidopsis, BR application did not significantly alter the expression of the tomato *ANT* ortholog during early ovary development, suggesting that BR-mediated regulation of ovule number in tomato may operate through distinct or *ANT*-independent pathways (Barro-Trastoy *et al*., 2020).

Altogether, both CK and BR are indispensable for reproductive success, acting through intricate and overlapping networks with auxin and other hormones to regulate fertilization, fruit set, and early fruit development. Their coordinated actions ensure precise control of cell division, tissue differentiation, and organ patterning, ultimately optimizing fruit yield and seed formation across diverse plant species.

## Jasmonic acid and salicylic acid in pollination and fertilization control

Pollination by insects is essential for the reproductive success of most flowering plants. In wild tobacco (*Nicotiana attenuata*), a functional genomic study demonstrated that JA plays a pivotal role in the production of nectar and benzyl acetone—two key factors that attract pollinators (Li *et al.*, 2018). Beyond pollinator attraction, JA has also been implicated in regulating pollen germination and pollen tube growth in diverse plant species, including rice and pine, although the underlying molecular mechanisms remain largely unresolved (Çetinbaş-Genç and Vardar, 2020; He *et al.*, 2021) (Fig. 1). In Arabidopsis, the F-box protein CORONATINE INSENSITIVE 1 (COI1), which mediates the perception of bioactive JA, is indispensable for pollen germination; loss-of-function *coi1* mutants are male sterile due to defective, non-germinating pollen (Xie *et al.*, 1998). Similarly, in tomato, the loss-of-function mutation in *JASMONIC ACID INSENSITIVE 1* (*JAI1*; an ortholog of Arabidopsis *COI1*) exhibits partial defects in pollen viability and germination (Li *et al.*, 2004; Dobritzsch *et al.*, 2015). In addition to defects in pollen viability and germination, the *jai1* mutant in tomato exhibits a pronounced female sterility phenotype, characterized by abnormal ovule morphology and impaired maturation, despite normal or near-normal carpel initiation (Schubert *et al.*, 2019). Transcriptomic analyses have revealed that the absence of JA signaling in *jai1* leads to widespread transcriptional reprogramming, with hundreds of genes differentially expressed. Notably, genes associated with cell wall modification, proteolysis (e.g. *METACASPASE9* and *SUBTILASE* genes), and nucleic acid degradation (e.g. *ENDONUCLEASE*) are significantly affected, particularly during later stages of ovule development. These molecular alterations correlate with increased callose deposition and vacuolation in the nucellus, indicative of disrupted cellular homeostasis and possibly premature or misregulated programmed cell death (Schubert *et al.*, 2019). Recent findings further reveal that COI1-dependent activation of the MYB21 TF acts as a positive regulator of reproductive success in both Arabidopsis and tomato (Song *et al.*, 2011; Niwa *et al.*, 2018; Schubert *et al.*, 2019). However, the downstream targets and precise molecular mechanisms through which MYB21 promotes reproductive development remain to be elucidated.

In addition to its role in ensuring pollen viability and proper ovule development, JA has also been found to play a significant part in post-pollination processes, including the regulation of fruit set and embryo development. In pumpkin (*Cucurbita pepo*), the JA biosynthetic enzyme LIPOXYGENASE 3 (LOX3) has been identified as a key regulator of fruit set (Cebrián *et al.*, 2023). LOX3 catalyzes the oxygenation of α-linolenic acid, initiating the JA biosynthetic pathway and leading to the accumulation of jasmonates, which are crucial for reproductive development (Wasternack and Hause, 2013). In pumpkin, fruit abortion frequently occurs in ethylene signaling mutants such as *aco1a* and *etr2b* due to impaired ethylene perception or biosynthesis. Remarkably, exogenous JA application can rescue this phenotype, indicating that JA can compensate for impaired ethylene signaling. JA up-regulates gene networks associated with cell division, expansion, and metabolic reprogramming in the ovary, processes that are essential for successful fruit initiation and growth (Cebrián *et al.*, 2023). The LOX3-dependent JA biosynthesis thus ensures adequate JA levels to trigger these developmental events, and exogenous JA can restore the hormonal balance required for fruit set even when ethylene signaling is compromised. The role of JA in reproductive success has been extensively studied in tomato. In the *spr2* mutant (mutation in a gene encoding a chloroplast ω-3 fatty acid desaturase responsible for JA biosynthesis), which is deficient in both 12-oxo-phytodienoic acid (OPDA; precursor of JA) and JA, embryo development is delayed and accompanied by increased programmed cell death in the developing seed coat and endosperm (Li *et al.*, 2003). In contrast, the *acx1a* mutant (mutation in a gene which encodes an acyl-CoA oxidase and functions at the first step of the β-oxidation phase in JA biosynthesis), which preferentially accumulates OPDA along with trace amounts of JA, exhibits normal embryo development comparable with the wild type, suggesting a functional role for OPDA in embryogenesis (Li *et al.*, 2005). The activity of residual JA in *acx1a* is considered unlikely, as the reproductive defects of the JA-insensitive *jai1* mutant can be rescued by wound-induced accumulation of OPDA (Goetz *et al.*, 2012). This hypothesis is further supported by *Allene Oxide Cyclase* (*AOC*)-RNAi plants (AOC enzyme catalyzes the unstable allene oxide intermediates into OPDA) which display embryo phenotypes similar to *jai1*, implicating a deficiency in OPDA biosynthesis (Goetz *et al.*, 2012); these findings suggest that OPDA or an OPDA-derived compound is required for proper embryo development, potentially by modulating carbohydrate allocation and detoxification processes. Besides JA biosynthesis, in strawberry (*Fragaria×ananassa*), JA signaling genes (e.g. *FaMYC2* and *FaJAZ1*) are highly expressed during early fruit development but decline during ripening, correlating with cell proliferation and fruit growth (Garrido-Bigotes *et al.*, 2018). This highlights the role of JA as a critical integrator of hormonal signals, coordinating the complex interplay between pollination, fertilization, and fruit development.

In addition to JA, SA also regulates pollen tube growth. After pollination, the SA content increases in apple and pear, which accelerates pollen germination and pollen tube growth, significantly boosting the fruit-setting rate (Lu *et al.*, 2021). Furthermore, a recent study using *Camellia oleifera* seed oil provided evidence that SA positively regulates pollen germination and pollen tube growth, possibly through the modulation of *NONEXPRESSOR OF PATHOGENESIS-RELATED* (*NPR*) genes (Lu *et al.*, 2021) (Fig. 2). Contrarily, in Arabidopsis, SA was found to regulate pollen tube growth through an NPR-independent pathway, probably through CME (clathrin-mediated endocytosis) function and the impact of Rho-like GTPase1 (ROP1) activity (Rong *et al.*, 2016) (Fig. 2).

Taken together, these findings underscore the multifaceted roles of JA and SA as key hormonal regulators in plant reproduction, not only orchestrating pollinator attraction and pollen performance but also ensuring successful fruit set through intricate hormonal crosstalk and molecular networks. Their ability to integrate and modulate developmental and environmental cues highlights their central importance in optimizing reproductive success across diverse plant species.

## Hormonal crosstalk during pollination, fertilization, and fruit set

**Fig. 3.**
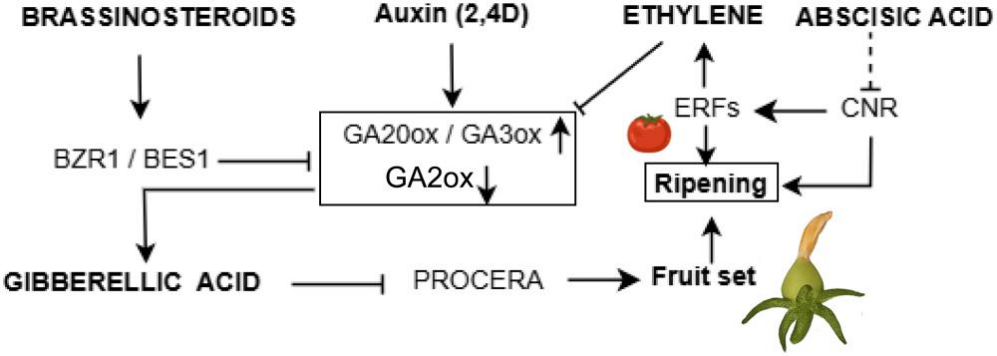
Schematic representation of crosstalk between different hormone signaling pathways in terms of fruit set and development primarily focusing on a fleshy fruit model such as tomato. In tomato, the brassinosteroid signaling component BRASSINAZOLE RESISTANT 1 (*BZR1*) suppresses gibberellin (GA) biosynthetic genes such as *GA20 oxidase* (*GA20ox*) and *GA3ox* to activate fruit set development. GA acts as a positive regulator in fruit set initiation through down-regulation of DELLA proteins (PROCERA in tomato), negative regulators of fruit development. The exogeneous application of auxin (2,4-D) enhances the expression of GA biosynthetic genes, showing a connection between these two hormones. The fine-tuning of abscisic acid (ABA) and ethylene enhances fruit set and delays senescence during early fruit set development through *COLORLESS NONRIPENING* (*CNR*), a key regulator of climacteric ripining. Moreover, CNR activates *ETHYLENE RESPONSE FACTOR* (*ERF*) genes and acts upstream of the ethylene-mediated ripening signaling cascades. Broken lines indicate indirect manipulation of a process or unknown mechanism. Activation is indicated with an arrow (→), while inhibition is indicated with a blunted arrow (┴). Created in BioRender. Panda, S. (2025) https://BioRender.com/mj44x7k.

Prominent examples are available that show interaction between diverse hormone pathways to regulate early events of fruit set (Fig. 2). Among these, auxin and GA play distinct yet interconnected roles in pollination, fertilization, and fruit set. In tomato, these hormones co-regulate intrinsic reproductive developmental processes through the crosstalk of their metabolic pathways (Gillaspy *et al.*, 1993; Raghavan, 2003; Fenn and Giovannoni, 2021). While the formation of bioactive GA in ovary tissue is reliant on the activities of GA20 and GA3 OXIDASES (GA20ox/GA3ox), auxin biosynthesis within the seed of Arabidopsis and tomato is predominantly regulated by the abundance of YUCCA (YUC) flavin monooxygenases and enzymes from the TRYPTOPHAN AMINOTRANSFERASE-RELATED (TAR) family that are known to be involved in auxin biosynthesis (Sun, 2008; Zhao, 2014; Pattison *et al.*, 2015) (Fig. 1). A similar mechanism has been observed in developing strawberry achenes, where co-induction of *YUC* and *GA20/3ox* gene expression supports the notion that auxin influences GA levels to facilitate fruit set (McAtee *et al.*, 2013; Kumar *et al.*, 2014). Expanding on this interaction, studies in tomato demonstrate that auxin enhances GA levels through up-regulation of GA biosynthetic genes (e.g. *GA20ox1/2/3*, *GA3ox1*, and *COPALYLDIPHOSPHATE SYNTHASE*) and down-regulation of GA catabolic genes (e.g. *GA2ox2*), resulting in increased levels of bioactive GA (Fig. 3). This mechanism is conserved in pear, where auxin application similarly stimulates GA biosynthesis and induces parthenocarpy (Cong *et al.*, 2019).

At the molecular level, Aux/IAA and ARF proteins were found to facilitate the intricate interaction between auxin and GA to maintain plant reproduction in Arabidopsis (Richter *et al.*, 2013) (Fig. 2). Several ARFs have been reported to repress the expression of GA biosynthetic genes (such as *GA20ox1* and *GA3ox1*) and consequently attenuate GA-related responses in both Arabidopsis and tomato (Fenn and Giovannoni, 2021). In tomato, ARF7 was found to interact with IAA9 and DELLA proteins, consequently inhibiting transcriptional activity of growth-inducing genes before fertilization (Hu *et al.*, 2018). The ARF7–IAA9 association blocked GA biosynthesis and auxin metabolism by suppressing expression of *GA20ox1/GA3ox1* and a *GRETCHEN HAGEN3 acyl adenylate/amidosynthetase* (*GH3.2*) gene [*GH3* genes coding for enzymes which lead to biosynthesis of phytohormone–amino acid conjugates through catalysis], hence preventing expansion during fruit set in tomato. Yet, during fertilization of tomato, the ovule releases auxin and GA, which triggers ubiquitination and degradation of IAA9 and DELLA. As a result, ARF7 is released to activate auxin- and GA-related responses (Hu *et al.*, 2018). Auxin and GA co-regulate several processes that support fruit set through coordination of Aux/IAAs and ARFs. In tomato, for example, IAA9 and IAA27 both mediate auxin sensitivity, while IAA27 is uniquely required for pollen and ovule fertility, and IAA9 controls genes promoting post-fertilization growth (Bassa *et al.*, 2012; Hu *et al.*, 2018) (Fig. 1). Additionally, besides the Aux/IAA-mediated regulation of auxin responses, the inactivation of free auxin molecules through conjugation is another way to regulate active levels of auxins. This process is facilitated by Group II GH3 proteins (GH3.1-11 and GH3.17), as repeatedly reported in numerous plants, including Arabidopsis, tomato, grapes, and rice (Staswick *et al.*, 2002, 2005; Böttcher *et al.*, 2010; Liao *et al.*, 2015; Wojtaczka *et al.*, 2022).

These Group II GH3 proteins are involved in the conjugation process between IAA and amino acids such as Glu, Ala, Asp, Gly, Val, and Leu. An exception is the AtGH3.5 enzyme, which accepts not only auxins but also benzoates as substrates (Staswick *et al.*, 2005; Westfall *et al.*, 2016). While CK is known to regulate the expression of Group II *GH3* genes in Arabidopsis roots (Pierdonati *et al.*, 2019), its co-regulatory role during fruit development remains to be elucidated.

An additional layer of hormonal complexity in reproductive development arises from the interplay among ethylene, auxin, and GA, which together orchestrate key processes such as male fertility, fertilization, and fruit set. In tomato, successful fertilization requires coordinated communication between ethylene and auxin signaling pathways, as demonstrated by their essential roles in pollen development and tube growth (Shinozaki *et al.*, 2015; Kovaleva *et al.*, 2017; An *et al.*, 2020). Ethylene further modulates reproductive transitions by antagonizing GA biosynthesis—specifically through down-regulation of *GA20ox* genes and stabilization of DELLA proteins, thereby attenuating GA activity. This mechanism has been reported in both tomato and citrus (Sharif *et al.*, 2022; Chu *et al.*, 2023) (Fig. 3). In tomato, disrupted ethylene perception leads to enhanced GA biosynthesis and response, resulting in parthenocarpic fruit development in the absence of fertilization (Kumar *et al.*, 2014; Shinozaki *et al.*, 2015). A similar hormonal repression is observed in non-pollinated watermelon flowers, where elevated ethylene and ABA levels are associated with up-regulation of DELLA and IAA9, key repressors of GA and auxin signaling, respectively—thus maintaining ovary dormancy until fertilization occurs (Hu *et al.*, 2018). The auxin–GA crosstalk has also been extensively studied in other fruit systems such as in strawberries. Here, early fruit development is modulated by the interaction between the GA signaling repressor RGA1 and the auxin-responsive ARF8 TF (Zhou *et al.*, 2021) (Fig. 1). ARF8 transcriptionally suppresses the GA receptor gene *GID1c* to fine-tune GA sensitivity, while simultaneously interacting with IAA4 to modulate auxin responses. Collectively, these findings reveal a sophisticated regulatory module in strawberry that integrates auxin and GA signaling to ensure proper progression of early fruit development.

In addition to the well-established roles of auxin and GA, crosstalk of GA and BRs has been shown in tomato, where BR suppresses the GA levels through the PROCERA–BZR1 complex which in turn facilitates the activation of ovule initiation gene regulators (Figs 1, 2) (Barro-Trastoy *et al.*, 2020). Interestingly, this interaction diverges in Arabidopsis, where the DELLA–BZR1 complex does not play a comparable role in ovule development, pointing to species-specific regulatory evolution (Barro-Trastoy *et al.*, 2020).

Other hormones including CK, JA, ABA, and ethylene further contribute to the intricate web of signaling during pollination, fertilization, and fruit set. Notably, investigations in tomato fruit development have revealed that CK-induced parthenocarpy is partly dependent on enhanced GA and auxin biosynthesis, emphasizing the multifaceted nature of hormonal regulation in this context (Ding *et al.*, 2013). In the case of fig (*Ficus carica*) flowers, an induction of key CK biosynthetic genes, including *NCED1* and *ACS*, has been observed, leading to a concurrent elevation in ABA and ethylene levels (Chai *et al.*, 2017), indicating a multi-hormonal convergence. Furthermore, the interconnection between ABA and ET has been hinted at through common TFs. ZINC FINGER TRANSCRIPTION FACTOR (*Sl*ZFP2) negatively regulates ABA biosynthetic genes to modulate tomato fruit development and delays ripining through the down-regulation of *COLORLESS NON-RIPENING*, a key ripening regulator that functions upstream of the ethylene-mediated ripening cascade (Weng *et al.*, 2015b) (Fig. 3). The evidence reflects the crosstalk of ABA and ethylene during fruit set and its maturation through the common TFs channeling different hormone signals in a specific development process.

Collectively, the initiation and progression of fruit set are governed by a dynamic and multilayered network of hormonal interactions. In addition to the well-established auxin–GA synergy and BR-mediated modulation of GA signaling, recent findings underscore more intricate crosstalk involving ethylene, ABA, and CKs. These hormones function in an integrated manner, aligning environmental signals with intrinsic developmental programs to ensure reproductive success. Gaining a deeper understanding of these interactions—particularly their spatial, temporal, and species-specific regulation—will be essential for pinpointing novel regulatory nodes during the early phases of fruit development that are amenable to targeted crop improvement strategies.

## Concluding remarks and perspectives

Despite significant advances in elucidating the hormonal regulation of fruit set and early fruit development, the molecular intricacies of hormone crosstalk and their integration with environmental cues remain only partially understood. Recent studies have highlighted the central roles of auxin–GA crosstalk, mediated by Aux/IAA and ARF TFs and further regulated by miRs such as miR160, miR167, and miR171, in orchestrating cell division, expansion, and the transition from fertilization to fruit set (Damodharan *et al.*, 2016; Fenn and Giovannoni, 2021; Godoy *et al.*, 2021; Li *et al.*, 2022). The involvement of ethylene in modulating auxin and GA levels during fertilization, along with emerging evidence for miR166-mediated regulation of *Sl*HB15A affecting auxin and ethylene signaling, further underscores the complexity of hormonal interplay (Clepet *et al.*, 2021).

Nevertheless, the precise mechanisms governing these interactions—especially those involving post-transcriptional and spatial regulation of hormone signaling—are still largely obscure. While the functional diversity of hormone transporters (such as ABCBs) and regulatory proteins (including GRAS family members) has been characterized in tomato, their conservation and roles in other fruit species remain to be fully explored (Huang *et al.*, 2017; Ofori *et al.*, 2018). Additionally, the contributions of other hormones such as CK, JA, and BR, as well as their integration with epigenetic modifications, represent promising but underexplored areas of research.

A key limitation in hormonal research is the difficulty of measuring hormone metabolites at cellular and tissue-specific resolution, due to their low abundance and rapid turnover. Although single-cell metabolomics has advanced considerably in both plant (Pandian *et al.*, 2023; Yu *et al.*, 2023) and animal (Wang *et al.*, 2025) systems, extending these techniques to single-cell hormonomics—focused on detecting individual hormone molecules—remains a major hurdle. This is primarily because, under non-stress conditions, hormone concentrations in specific cells or tissues are often too low and typically fall below the sensitivity threshold of current analytical platforms (Vrobel and Tarkowski, 2023). Addressing this challenge will require the development of ultra-sensitive detection methods, such as next-generation MS and real-time biosensors, to enable high-resolution mapping of hormone dynamics during critical developmental processes. Equally important is the need to understand how hormonal networks maintain reproductive stability and fruit set under abiotic stress, especially in the face of climate change. Hormones such as CK, auxin, and ABA play central roles in balancing the trade-off between plant survival and reproduction, directly influencing processes such as gametophyte development, fertilization, and fruit initiation, especially under heat and drought stress (Kurepa and Smalle, 2023; Begcy *et al.*, 2024). Recent research highlights that reproductive organs, including pollen and ovules, are particularly sensitive to elevated temperatures, leading to reduced fertility and fruit set if hormonal regulation is disrupted (Raza *et al.*, 2019; Begcy *et al.*, 2024). Furthermore, future efforts should prioritize the use of genome editing and precision breeding to fine-tune hormone biosynthesis and signaling pathways for enhanced crop resilience. For example, in maize, CRISPR/Cas9 [clustered regularly interspaced palindromic repeats (CRISPR)/CRISPR-associated protein 9]-mediated promoter editing of the ethylene signaling repressor *ARGOS8* reduced ethylene sensitivity and significantly improved grain yield under drought by enhancing reproductive success and seed set (Shi *et al.*, 2017). Similarly, in tomato, CRISPR/Cas9-mediated knockout of *Sl*AGL6 and *Sl*ARF8a, which are functionally linked to the auxin–GA and aux–JA signaling pathways, respectively, resulted in parthenocarpic fruit development under heat stress (Klap *et al.*, 2017; Israeli *et al.*, 2023). This finding highlights that targeted manipulation of hormone-responsive genes can help maintain fruit set when pollination is compromised by elevated temperatures.

Looking forward, integrative, high-resolution strategies combining multi-omics, single-cell transcriptomics, and spatial metabolomics will be crucial for decoding hormone action at the cellular level. Simultaneously, dissecting the contributions of chromatin remodeling, small RNAs, and non-coding RNAs will offer insights into the epigenetic and post-transcriptional control of hormonal signaling. Ultimately, integrating these diverse data layers into predictive systems biology frameworks will accelerate the identification of key regulatory hubs, informing the rational design of climate-resilient, high-yielding crops.

In summary, a deeper mechanistic understanding of hormone crosstalk, regulatory networks, and their environmental integration will be pivotal for advancing both basic plant science and applied crop improvement. By leveraging new technologies and interdisciplinary approaches, future research can unlock strategies for stable fruit set and yield, ensuring food security and sustainability in the face of global challenges.
